# The Current and Future States of Diagnostic Tests for Histoplasmosis with a Focus on People with HIV and Disseminated Histoplasmosis

**DOI:** 10.3390/jof9080793

**Published:** 2023-07-28

**Authors:** Kenneth Villareal, Austin Price, Alessandro C. Pasqualotto, Nathan C. Bahr

**Affiliations:** 1 Division of Infectious Diseases, Department of Medicine, University of Kansas Medical Center, Kansas City, KS 66160, USA; kvillareal@kumc.edu (K.V.); aprice3@kumc.edu (A.P.); 2 Department of Clinical Medicine and Post-Graduation Program in Pathology, Universidade Federal de Ciências da Saúde, Porto Alegre 90050-170, Brazil; acpasqualotto@gmail.com

**Keywords:** histoplasmosis, *Histoplasma capsulatum*, diagnostic tests, disseminated histoplasmosis, endemic mycoses, HIV/AIDS

## Abstract

Histoplasmosis is caused by *Histoplasma capsulatum* and, although endemic in large parts of the world, is often underrecognized in many locations. In addition to underrecognition, inadequate availability of diagnostic tests is a major contributor to poor outcomes in disseminated disease in people with HIV. For those with advanced HIV and disseminated disease, antibody testing is less useful. Culture and histopathology can be useful in this situation, but each has limitations, including variable sensitivity by site and, in the case of culture, the need for a biosafety level three laboratory and a long period of growth. Antigen testing has proven useful for disseminated histoplasmosis due to the excellent sensitivity of urine. Yet, turnaround is slower than ideal due to use in a limited number of centers. The development of lateral flow assays has the potential to make for true rapid point-of-care assays for histoplasmosis, but in order to meet that promise, the tests must be widely available and affordable.

## 1. Introduction

Histoplasmosis is caused primarily by *Histoplasma capsulatum* var. *capsulatum* but also, less commonly, by *Histoplasma capsulatum* var. *duboisii* [1]. At least 500,000 cases are estimated to occur annually in the United States alone, although the real number is likely much higher [1]. Traditionally, histoplasmosis has been taught to be endemic primarily in the Ohio and Mississippi River valleys within the United States and large parts of South and Central America [2]. It has become increasingly clear in recent decades that, in fact, histoplasmosis occurs in much of the world [2]. Yet, there are some areas where histoplasmosis is much less common (much of northern Africa and Europe) and other areas that continue to have much higher rates of histoplasmosis (such as the Central United States and Latin America) [2]. In Central and South America, for instance, histoplasmosis is as common as TB among those with HIV [3]. A major reason for our improved understanding is that the HIV pandemic has ‘unmasked’ cases of histoplasmosis in many parts of the world where they were not previously known to occur [4]. Similarly, increased use of immune suppressing medications has led to increased cases in many locations [4]. Although our understanding of regional endemicity is improving, it is clearly still incomplete—*Histoplasma capsulatum* was recently detected on Antarctica, for instance [5]. Ultimately, improving our knowledge of the endemicity of *Histoplasma capsulatum* is crucial for correct diagnosis. If the clinician does not consider histoplasmosis, the diagnosis will usually be missed. Yet, for this knowledge to be maximally beneficial to patients, diagnostic testing must be accurate, rapid, and widely available.

Diagnostic certainty is of particular importance in disseminated histoplasmosis, which is uniformly fatal if not treated [6]. Traditional diagnostic techniques are slow (culture) and/or of variable sensitivity (direct visualization). Antigen detection tests are fairly rapid and sensitive, but they have not generally been widely available outside of the United States.

In this review, we will discuss the current state of diagnostic testing for histoplasmosis. We will further discuss diagnostic test performance differences between people with and without HIV. We will also discuss newer tests that are expected to be commercially available in the near future, are currently being tested, or are expected to begin testing in the coming months to years. Further, we will discuss the potential effects that these tests might have on the diagnostic landscape of histoplasmosis and the steps necessary to make certain that these tests have the highest possible impact for patients.

## 2. Current State of Diagnostics

There are a number of different techniques used in the diagnosis of histoplasmosis. The diagnostic accuracy of each microbe-focused modality varies by syndrome and immune status because they affect the fungal burden. Antibody test performance is also affected by immune response, given that it is a test of the immune response to histoplasmosis. Thus, it is important to specify this when discussing histoplasmosis diagnostics [7]. Histoplasmosis pulmonary disease can be acute, subacute, or chronic [8]. Disseminated disease, pulmonary nodules, lymphadenopathy, and other forms occur as well [9]. Disseminated disease implies the spread of histoplasmosis beyond the pulmonary system into the blood and other organs [7,8]. This syndrome often occurs in immunocompromised patients or those with a large inoculum load leading to infection [7,8]. In addition to an appropriate clinical syndrome, the diagnosis of histoplasmosis can be made or supported via culture, pathological evaluation, antigen and/or antibody testing, and polymerase chain reaction (PCR) [7].

Traditionally, growth of *Histoplasma* on culture from a clinical specimen has been the gold standard for diagnosis [10]. Despite this, the overall sensitivity of culture is quite low for some types of histoplasmosis (50 to 85 percent) and varies widely [11]. Culture can be performed on a number of specimens, including sputum, blood, bone marrow, or almost any tissue suspected to be involved [1]. Growth on standard blood culture media is rare and generally does not occur within the 5-day period for which most cultures are monitored. Specific laboratory techniques can improve culture yield. For example, lysis-centrifugation of blood samples has been shown to have superior sensitivity to that of automated systems, and the addition of ammonium hydroxide to the culture media of respiratory samples suppresses the growth of commensal organisms, allowing for enhanced growth of *Histoplasma* [12,13,14,15]. Bone marrow or blood cultures are most useful for disseminated disease, where sensitivity is ~75% [1]. In pulmonary disease, sputum or bronchoscopy cultures may be helpful. However, these cultures are most useful in sub-acute or chronic pulmonary disease and generally less useful in acute pulmonary disease [7]. Notably, culture takes at least four weeks for growth, which may limit clinical utility in some cases.

Direct visualization is useful in many cases, especially when positive. *Histoplama capsulatum var. capsulatum* yeast are ovoid and 2–4 µM, while *Histoplama capsulatum var. duboisii* are larger, generally 6–12 µM [1]. Given the rapid information obtained and relatively inexpensive cost of these tests, they are likely the most commonly utilized test type worldwide for histoplasmosis. Respiratory specimens (particularly for people with sub-acute or chronic pulmonary disease), bone marrow (for those with disseminated histoplasmosis), and tissue specimens (from the site of infection) are the most frequent sites from which histopathologic testing yields results [1,7]. Sensitivities can vary widely (9–50%) depending on tissue type and disease syndrome [9,16,17,18,19]. Importantly, tissue or lung specimens may show yeast consistent with *Histoplasma* in active infection or prior infection, and so clinical interpretation is crucial [7]. Sensitivity of culture and direct visualization generally improves with more advanced HIV as a lesser immune response allows for a higher fungal burden, and so there is a higher likelihood of growing fungi or visualizing fungi [20].

Antigen testing is available on blood and urine in most cases and can be performed on cerebrospinal fluid and bronchoalveolar lavage (BAL) specimens as well. Antigen testing performs well for disseminated disease and acute pulmonary disease, particularly in the context of advanced HIV [1,18]. Antigen testing is less sensitive in chronic pulmonary histoplasmosis due to the lower availability of peripheral antigen to be detected [9]. This modality is at risk of false positives due to the cross-reactivity of other fungal pathogens such as *Coccidioides* and *Blastomyces* species. Antigen testing will be further discussed below in the context of new tests being developed that may drastically change antigen test accessibility.

A variety of antibody tests are available for histoplasmosis, including immunodiffusion, complement fixation, and enzyme immunoassays. Immunodiffusion test results include H bands (which generally clear within six months of infection and are present in <25% of cases) and M bands (present in ~75% of cases but which persist for years) [1,21,22]. Thus, the interpretation of H bands is more straightforward when no recent histoplasmosis is suspected, but M bands may represent old exposure when found in isolation. Complement fixation testing gives titers. Most patients with histoplasmosis have a titer of at least 1:8 after a month or two of illness, but positive titers can linger for years. Titers of 1:32 or higher are more consistent with active infection [1]. Antibody testing offers higher sensitivity for subacute pulmonary histoplasmosis and chronic pulmonary histoplasmosis but is less reliable in testing for acute pulmonary histoplasmosis and early disseminated disease as it may take weeks for sufficient numbers of antibodies to develop [20,23]. Antibody tests can also be subject to false positives related to infection with other fungal pathogens [20]. Patients with depressed immune responses, particularly those with immune suppression due to solid organ transplantation, may not produce expected antibody responses and so may test negative despite active infections [7,8,24]. In addition, many physicians are not experienced in interpreting *H capsulatum* antibody testing, and so may not properly use the test results [1]. This is a crucial point because many of the antibody tests described require an understanding of the clinical context and other diagnostic tests in order to correctly interpret the antibody test in question. For instance, a complement fixation titer of 1:16 is not an uncommon scenario but may be interpreted quite differently in a person with a positive urine antigen test and sputum culture compared to a person in whom those other tests are negative. Further, that same scenario with a single positive complement fixation test at 1:16 (in the setting of a negative urine antigen and sputum culture) might be seen quite differently in a person with an isolated pulmonary nodule versus a person with a syndrome consistent with acute pulmonary histoplasmosis.

PCR has great potential, but implementation has been inconsistent. Sensitivity can be high in PWH and disseminated disease, but the lack of protocol and target standardization has limited utility and its role in diagnosing histoplasmosis is not clear [25,26]. Further, even in people without HIV, PCR can be utilized in targeted specimen types where infection is suspected, such as respiratory specimens [27]. One study used PCR on BAL specimens, but it was positive in only two of six samples that were positive by culture [28]. In other studies, the sensitivity was similarly poor in urine, serum, and CSF [29,30]. For both people with and without HIV, further study and standardization are needed. Further work is needed on defining the molecular target given the current lack of consensus [7,31,32].

## 3. Performance of Diagnostic Tests for People with Disseminated Histoplasmosis with and without HIV

Given the broad landscape of diagnostic tests available for histoplasmosis, one must keep in mind a number of factors that may affect test performance, including suspected syndrome, specimen type, resource availability, and the immune status of the source patient. All these factors affect the performance and, thereby, the diagnostic accuracy of any given test [1,7,9,33]. For the purposes of this review, we are particularly interested in how HIV infection might affect diagnostic test accuracy, keeping in mind that the degree of immune suppression (and so diagnostic test performance) may vary amongst people with HIV (PWH). HIV status is particularly important given the high incidence of histoplasmosis in PWH in many countries [3,34].

In disseminated disease, cultures may be obtained from nearly any site where infection is suspected, although bone marrow and blood cultures are most useful (overall sensitivity ~75%) [1,9]. Notably, cultures from the cerebrospinal fluid are of particularly low yield. One study from a Brazilian referral center found that among 99 evaluable patients diagnosed with histoplasmosis, 59 had culture performed. Of those, the sensitivity was 25% (5/20) in people without HIV and 71.7% (28/39) in PWH, *p* < 0.01 [35]. This included blood culture and bone marrow culture, where sensitivities were 32.3% and 36.9% in PWH and 11.8% and 8.8% in people without HIV (*p* 0.025 and 0.003), respectively [35]. Antigen testing was not available in this report. Another study of only PWH found 33.7% sensitivity (33/98) among 98 people with disseminated histoplasmosis where antigen testing was available [36]. In that study, in Guatemala and El Salvador, the median CD4 count was 29 cells/µL, only 37% were on anti-retroviral therapy at diagnosis, and antigen (CDC ELISA) sensitivity was 86.7% (85/98) [36]. Thus, it is likely that the true diagnostic yield of culture depends on the case definition, including which tests were available in the center to make up the underlying cases. Lastly, in a study of 349 PWH in French Guiana with disseminated histoplasmosis, 214 had at least one culture taken [37]. Liver biopsy yielded 86.9% sensitivity (53/61), lymph node 79.6% (43/54), bone marrow 77.6% (156/201), and lower digestive tract samples 74% (40/54) [37]. Cultures of skin lesions are usually positive, but this represents a late diagnosis. Thus, not only does performance vary by HIV status but also by specimen type.

Histopathologic diagnosis of *Histoplasma* by direct microscopy is possible in patients with disseminated disease. Specimens obtained from the bone marrow, liver, skin, and mucous membranes often reveal organisms when stained with methenamine silver or periodic-acid Schiff stains [20]. In the same Brazilian study noted above, histopathology sensitivity was 58.3% (7/12) among people without HIV and 72% (18/28) among PWH [35].

Peripheral blood smears may show yeast forms within circulating macrophages in severe disseminated infection, but the sensitivity is exceedingly low (less than 10%) [20,38]. As with culture, histopathologic diagnosis requires skilled personnel to prepare and interpret the specimen, which can be limited in certain settings. The combined use of culture and direct microscopy increases diagnostic sensitivity. One study including 36 patients with advanced HIV reported a diagnostic sensitivity of 88 percent when blood culture and direct microscopy of peripheral blood smears were used concomitantly [38]. In French Guiana, among PWH, only 32 of 214 (14.9%) bone marrow specimens were sent for cytopathology, and lower numbers were sent for BAL and CSF specimens [37]. Cytopathology sensitivity was 34.4% (11/32) for bone marrow, 50% (5/10) for BAL, and 0% (0/5) for CSF [37]. Pathology utilization was higher in general: 72.9% (43/59) for the lower digestive tract, 66.1% (39/63) for the lymph node, and 50.7% (38/75) for the liver [37]. Direct examination (rapid examination by mycologists shortly after they are obtained, as opposed to standard pathology staining) was utilized frequently, particularly for bone marrow and BAL (both > 97% of samples). Sensitivity ranged from 73.7% (28/38) for mucocutaneous samples to 34.6% (72/208) for bone marrow samples [37]. The message from Nacher and colleagues was clear: although test performance may vary by tissue/fluid type, diagnosis attempts via this method are underutilized, particularly where antigen testing is not available [37].

Serologic antibody testing for *Histoplasma* is unreliable in disseminated disease. This is related to the immunocompromised status of the host, whether from chronic immunosuppressive drugs or from conditions that are deleterious to the cellular immune system, particularly HIV/AIDS, both of which impair antibody formation [39]. Modern antibody detection includes both complement fixation (CF) and immunodiffusion (ID) assays and Western blotting. In advanced HIV patients, the combined use of CF and ID assays produces a variable diagnostic yield of 70 to 92 percent [18,40]. ID assay is less sensitive (approximately 80%) but more specific than CF, particularly in disseminated infections when the presence of the generally absent H-band is most likely to be seen [40,41]. The study from Brazil noted 65% (39/60) sensitivity among PWH and 83.8% sensitivity in people without HIV for immunodiffusion [35]. These investigators also found ~90% sensitivity for Western blot among those with and without HIV [35]. Of the seven cases falsely negative by Western blot, six were among PWH [35]. Another Brazilian study found 25% sensitivity by immunodiffusion among 12 PWH and 12.5% among eight people without HIV [42]. One meta-analysis including five studies found a sensitivity of 58% (95% CI 53–62%) for antibody detection assays of various types for disseminated histoplasmosis in PWH [25].

PCR is not commonly used clinically in some centers, but in other centers, it is a frequently utilized technique. PCR use has been reported among 12 PWH (all with disseminated disease) and 8 people without HIV (a mix of disseminated disease as well as chronic, sub-acute, and acute pulmonary histoplasmosis) in one study from Brazil, as well as in some other, smaller studies [42,43]. Dantas and colleagues found variable PCR performance by primer, finding 91.6% sensitivity using blood from PWH and the HC5.8S-ITS primer (33–66% with other primers) and 37.5–50% sensitivity, varying by primer, in people without HIV [42]. In one meta-analysis of five studies, the overall sensitivity was 95% (95% CI 89–100%) and the specificity was 99% (95% CI 96–100%) [25]. Yet, each study examined performance on different sample types (bone marrow, blood, tissue, respiratory samples, etc.), and each used different PCR protocols and/or gene targets; all were in-house tests [25]. To date, there is no commercially available PCR test for the detection of histoplasmosis. PCR protocols are not yet standardized, which is a big limitation. Yet, a multicenter external quality assessment has been recently published, and although samples were few, this was really seen as a potential first step towards standardization [26].

Antigen testing is the most sensitive modality for diagnosing disseminated histoplasmosis [18,25,44]. Samples from the serum, urine, CSF, and BAL fluid can be sent for antigen detection [44]. There are several commercially available antigen tests that are highly sensitive for disseminated disease. In PWH, antigen detection is particularly robust, with antigenuria being the most sensitive, followed by antigenemia [45]. The performance of various Histoplasma antigen tests on urine (including those not yet commercially available) is summarized in Table 1. One systematic review found that among 13 studies, antigen detection was 95% sensitive (95% CI 94–97%) among PWH with disseminated histoplasmosis [25]. This study included multiple different types of antigen assays. Performance in immunocompromised people without HIV is still excellent (93.1%, 81/87 in one study) but less sensitive (73.3%, 11/15) among people without known immune compromise [18]. Antigen testing is further discussed below.

## 4. Current and Future State of Antigen Testing and Disseminated Histoplasmosis

*Histoplasma* antigen detection has revolutionized the diagnosis of disseminated histoplasmosis, given its excellent performance and the ability to test non-invasive samples such as urine [54]. The performance of the first-generation quantitative sandwich enzyme immunoassay assay was published by Wheat and colleagues in the 1980s [46]. High levels of *Histoplasma capsulatum* polysaccharide antigen were detected in the urine of 97% of patients with disseminated histoplasmosis. Cross-reactions were described with other endemic mycoses, including blastomycosis, sporotrichosis, paracoccidioidomycosis, penicilliosis (talaromycosis), and coccidioidomycosis [47]. The second generation of the MiraVista test reduced false positives from the previous method [55]. Among 56 cases of disseminated disease in PWH, sensitivity was 94.6% (53/56) [18]. Since these assays were only performed at the MiraVista Diagnostics headquarters in Indianapolis, IN, USA, they were of very limited use in other parts of the world where histoplasmosis is endemic.

IMMY (Norman, OK, USA) then developed the first commercial test to detect *Histoplasma* antigen that could be used outside of the USA. The assay (IMMY ALPHA ELISA kit), a two-step sandwich-type immunoenzyme assay using polyclonal antibodies, was validated in urine samples in 2007 by Cloud and colleagues [56]. Cross-reactions also occurred with other endemic mycoses. Overall agreement between MiraVista and IMMY was high at 98% in disseminated histoplasmosis, even though the IMMY test was found to be less sensitive than the MiraVista test [57,58].

The U.S. Centers for Disease Control and Prevention (CDC) developed an in-house enzyme-linked immunosorbent assay (ELISA) for *H. capsulatum* detection. This assay identified *H. capsulatum* antigens using polyclonal antibodies with 81% sensitivity and 95% specificity, according to a study conducted in Guatemala [59]. In Colombia, the CDC test showed 71% sensitivity and 86% specificity when performed on serum samples [60]. Cross-reactions also occurred in patients with paracoccidioidomycosis. In Brazil, the CDC and the IMMY tests were concordant in 96% of cases [61]. Each of these studies involved PWH with disseminated histoplasmosis. Years later, the CDC stopped producing their *Histoplasma* antigen detection test.

IMMY subsequently launched a *Histoplasma* antigen-detection test based on monoclonal antibodies (Clarus assay) that showed improved sensitivity (95%) in comparison to the previous polyclonal antibody-based assay among PWH and culture-confirmed histoplasmosis [49]. In a study of 415 PWH, 108 persons had proven disseminated histoplasmosis by culture or histopathologic examination. Using 391 urine samples, the prior IMMY ALPHA showed 67.3% (95% CI 57.4–76.2%) sensitivity and 96.2% (95% CI 93.2–98.0%) specificity compared to 91.3% (95% CI 84.2–96.0%) sensitivity and 90.9% (95% CI 88.5–95.1%) specificity for IMMY Clarus [50].

Recently, multiple manufacturers have developed lateral flow assays (LFAs) for antigen detection. This type of technology is ideal in that it can generally be made relatively cheaply, does not require significant infrastructure, and can be performed in a rapid, decentralized fashion. MiraVista’s LFA has shown excellent performance in serum among 24 people with culture proven disseminated histoplasmosis and 51 controls (sensitivity 96%, specificity 90%) [62]. In the same study among Mexican PWH referenced above with the IMMY tests, the MiraVista LFA showed 90.4% (95% CI 83.0–95.3%) sensitivity and 92.3% (95% CI 88.6–95.1%) specificity, very similar to the IMMY Clarus EIA [50]. Using urine samples from PWH (26 with disseminated histoplasmosis and 74 controls), the MiraVista LFA showed equal sensitivity (96%) compared with the MiraVista EIA but with better specificity (96% vs. 77%) [51]. The MiraVista LFA has also been tested using urine specimens from 352 people, including 66 with proven or probable histoplasmosis (71% immunocompromised (HIV vs. other not noted), 46 disseminated, and 20 pulmonary) [52]. In this population, the sensitivity was 78.8% (52/66) and significantly less sensitive than the MiraVista EIA (95.5% (63/66), *p* 0.009) [52]. As would be expected, the test was only 50% sensitive (10/20) for pulmonary disease compared to 91.3% (42/46) for disseminated disease.

**Figure 1:**
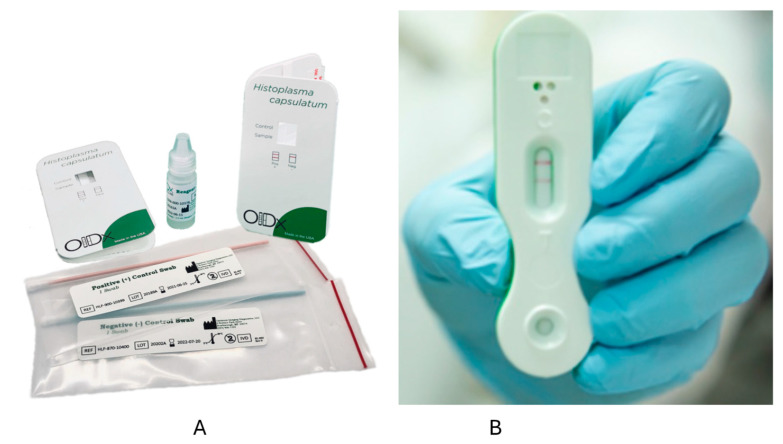
Lateral flow assays from OIDx (**A**) and MiraVista (**B**). The left figure (**A**) shows the kit for the OIDx lateral flow assay [64]. On the right is a positive test using the MiraVista lateral flow assay (**B**) [65].

Optimum Imaging Diagnostics (OIDx) has also developed an LFA. In one study from Ghana, PWH had urine samples collected and tested via the OIDx LFA and the IMMY Clarus EIA [63]. Among 107 participants, five cases were detected by the IMMY EIA, and six were positive by the OIDx LFA, including those positive by the IMMY EIA [63]. The OIDx LFA was also positive in two of 75 controls [63]. One other report described positive results in four PWH suspected to have histoplasmosis in Cameroon [64]. In another study using urine samples from 78 PWH, including 25 with disseminated histoplasmosis and 53 controls (including 10 from people without HIV), sensitivity was 92% (23/25), but specificity was only 32% (17/53) [53]. The OIDx and Mira Vista LFAs have European Union CE marks but are not FDA-approved (Figure 1). IMMY is also developing an LFA, but this has not been tested on clinical samples to our knowledge.

*Histoplasma* antigen is now seen as the standard of care for AIDS patients suspected of having disseminated histoplasmosis. In 2019, the World Health Organization (WHO) endorsed *Histoplasma* antigen testing as an essential diagnostic test [66]. In the same year, an international group of experts in Uganda strongly recommended *Histoplasma* antigen testing as an essential in vitro diagnostic for advanced HIV disease [67]. In a meta-analysis, the overall sensitivity and specificity of *Histoplasma* antigen detection in disseminated histoplasmosis in AIDS were 95% and 97%, respectively [25]. Accordingly, in the latest WHO guidelines for histoplasmosis, authors stated that “among people living with HIV, disseminated histoplasmosis should be diagnosed by detecting circulating *Histoplasma* antigens” [68]. However, most countries in Africa and Latin America still do not have access to the tests, mostly due to cost-related issues [69,70,71].

## 5. Conclusions

The diagnosis of histoplasmosis remains complicated, influenced by clinical syndromes and host immune status. In many settings, culture and histopathological examination remain the cornerstone of the diagnosis, but with significant limitations and barriers. PCR testing is used in some laboratories but requires further study and standardization. Antigen testing is now accepted as the preferred diagnostic technique for disseminated histoplasmosis and is particularly effective in those with HIV and high fungal burdens. Current antigen tests utilizing laboratory-based procedures cannot truly be point-of-care and are often limited by laboratory capacity and throughput. Thus, the development of rapid, point-of-care lateral flow assays is an exciting development. If these tests are indeed highly accurate, rapid, and easy to use, they will also need to be widely available worldwide and affordable in order to fully realize their potential. In PWH, particularly advanced HIV, much of the burden of histoplasmosis is disseminated disease, which means poorer outcomes compared to pulmonary disease alone. The availability of widespread, affordable, and accurate LFAs would drastically improve our understanding of the true burden of disease and would likely improve outcomes as well.

## Figures and Tables

**Table 1:** Available data on the performance of urine antigen testing in disseminated histoplasmosis *.

Test	Sensitivity	Specificity **	Population	Currently Commercially Available
**MiraVista EIA** [18,46,47,48]	91–100%	99%	People with HIV	Yes
**MiraVista EIA** [18,48]	93–94%	99%	People with other immune compromises	Yes
**MiraVista EIA** [18,48]	64–73%	99%	People without immune compromises	Yes
**IMMY Clarus EIA** [49,50]	91–95%	91–97%	People with HIV	Yes
**MiraVista LFA** [50,51]	90–96%	92–96%	People with HIV	No
**MiraVista LFA** [52]	91%	99%	People with HIV, people with other immune compromises, people without immune compromises	No
**OIDx LFA** [53]	92%	32%	People with HIV and people without HIV	No

* This is a summary of some available evidence and is not meant to be inclusive of all available data (e.g., a systematic review and meta-analysis were not performed). ** Specificity is high in healthy controls, but cross-reactivity is common in people with other fungal infections, particularly blastomycosis.

## Data Availability

This review created no new data and so no data is available from the review.
